# Equipment in anaesthesia and critical care

**DOI:** 10.1186/cc13743

**Published:** 2014-02-25

**Authors:** Bernd Saugel, Daniel A Reuter

**Affiliations:** 1 Department of Anesthesiology, Center of Anesthesiology and Intensive Care Medicine, University Medical Center Hamburg–Eppendorf, Martinistrasse 52, 20246 Hamburg, Germany

## 

Daniel Aston, Angus Rivers, Asela Dharmadasa: *Equipment in Anaesthesia and Critical Care.* Scion Publishing Limited, 2014, 404 pp., ISBN 978-1-907904-05-9.

In *Equipment in Anaesthesia and Critical Care* authors Daniel Aston, Angus Rivers, and Asela Dharmadasa aim to present a structured overview of basic underlying principles, mechanics, physics, and clinical application of technical equipment used in anaesthesia and intensive care medicine. According to the authors, the book is primarily intended for physicians preparing for the Fellowship of the Royal College of Anaesthetists examination.

Numbering 404 pages, this textbook offers a comprehensive overview in 13 chapters covering the technical background of most of the equipment used in daily clinical practice in the fields of anaesthesia and critical care. While Chapters 1 to 8 are dedicated to explaining equipment used in perioperative anaesthetic care (medical gases, airway equipment, breathing systems, ventilators, delivery of anaesthetic agents, monitoring equipment, filters and humidifiers, regional anaesthesia), Chapter 9 covers aspects of intensive care medicine including – among others – intravenous lines, monitoring equipment, and extracorporeal organ support. In addition, there are chapters on surgical equipment relevant to anaesthetists and radiological equipment used in the care of surgical or ICU patients. At the end of the book, the authors present several sample Fellowship of the Royal College of Anaesthetists questions.

Using a pragmatic approach with a standardised format for each section of the different chapters explaining the different pieces of medical equipment, the authors have been able to present a concise, well-arranged, and easy-to-read textbook giving an excellent overview of medical devices used in the operating theatre and the ICU. Throughout the textbook, each section is uniformly arranged as follows: Overview, Uses, How it Works, Advantages, Disadvantages, and Safety. The book is very richly illustrated with photographs and coloured illustrations explaining the equipment, as well as diagrams and line drawings helping to understand basic technical principles, normal values, and clinical application of the devices.

While *Equipment in Anaesthesia and Critical Care* gives an excellent, in-depth overview of technical and mechanical principles of the different devices, clinical applications and pathophysiologic considerations are not the primary scope of this technical textbook. In addition, the book could be even more informative for the academic reader if the authors provided references to scientific articles on the different devices.

Altogether, this textbook offers an excellent detailed overview of basic principles, mechanics, and physics of technical equipment used in anaesthesia and intensive care medicine. *Equipment in Anaesthesia and Critical Care* will therefore not only be very informative for physicians preparing for the Fellowship of the Royal College of Anaesthetists examination, but also for anaesthetists and intensivists aiming to understand the technical background of devices frequently used in their field.

## Competing interests

The authors declare that they have no competing interests.

